# Coronavirus Disease 2019 (COVID-2019) Infection Among Health Care Workers and Implications for Prevention Measures in a Tertiary Hospital in Wuhan, China

**DOI:** 10.1001/jamanetworkopen.2020.9666

**Published:** 2020-05-21

**Authors:** Xiaoquan Lai, Minghuan Wang, Chuan Qin, Li Tan, Lusen Ran, Daiqi Chen, Han Zhang, Ke Shang, Chen Xia, Shaokang Wang, Shabei Xu, Wei Wang

**Affiliations:** 1 Department of Hospital Infection Management, Tongji Medical College, Tongji Hospital, Huazhong University of Science and Technology, Wuhan, China; 2 Department of Neurology, Tongji Medical College, Tongji Hospital, Huazhong University of Science and Technology, Wuhan, China; 3 Beijing Infervision Technology, Beijing, China

## Abstract

**Importance:**

Health care workers (HCWs) have high infection risk owing to treating patients with coronavirus disease 2019 (COVID-19). However, research on their infection risk and clinical characteristics is limited.

**Objectives:**

To explore infection risk and clinical characteristics of HCWs with COVID-19 and to discuss possible prevention measures.

**Design, Setting, and Participants:**

This single-center case series included 9684 HCWs in Tongji Hospital, Wuhan, China. Data were collected from January 1 to February 9, 2020.

**Exposures:**

Confirmed COVID-19.

**Main Outcomes and Measures:**

Exposure, epidemiological, and demographic information was collected by a structured questionnaire. Clinical, laboratory, and radiologic information was collected from electronic medical records. A total of 335 medical staff were randomly sampled to estimate the prevalence of subclinical infection among a high-risk, asymptomatic population. Samples from surfaces in health care settings were also collected.

**Results:**

Overall, 110 of 9684 HCWs in Tongji Hospital tested positive for COVID-19, with an infection rate of 1.1%. Of them, 70 (71.8%) were women, and they had a median (interquartile range) age of 36.5 (30.0-47.0) years. Seventeen (15.5%) worked in fever clinics or wards, indicating an infection rate of 0.5% (17 of 3110) among first-line HCWs. A total of 93 of 6574 non–first-line HCWs (1.4%) were infected. Non–first-line nurses younger than 45 years were more likely to be infected compared with first-line physicians aged 45 years or older (incident rate ratio, 16.1; 95% CI, 7.1-36.3; *P* < .001). The prevalence of subclinical infection was 0.74% (1 of 135) among asymptomatic first-line HCWs and 1.0% (2 of 200) among non–first-line HCWs. No environmental surfaces tested positive. Overall, 93 of 110 HCWs (84.5%) with COVID-19 had nonsevere disease, while 1 (0.9%) died. The 5 most common symptoms were fever (67 [60.9%]), myalgia or fatigue (66 [60.0%]), cough (62 [56.4%]), sore throat (55 [50.0%]), and muscle ache (50 [45.5%]). Contact with indexed patients (65 [59.1%]) and colleagues with infection (12 [10.9%]) as well as community-acquired infection (14 [12.7%]) were the main routes of exposure for HCWs.

**Conclusions and Relevance:**

In this case series, most infections among HCWs occurred during the early stage of disease outbreak. That non–first-line HCWs had a higher infection rate than first-line HCWs differed from observation of previous viral disease epidemics. Rapid identification of staff with potential infection and routine screening among asymptomatic staff could help protect HCWs.

## Key Points

**Question:**

What are the exposure details and clinical characteristics of health care workers with coronavirus disease (COVID-19) in Wuhan, China?

**Findings:**

In this single-center case series including 9684 health care workers, 110 of whom had COVID-19, a higher rate of infection was found among those working in the low-contagion area during the early stage of the disease outbreak, especially among nurses younger than 45 years. Most health care workers with COVID-19 had nonsevere disease, with an asymptomatic carrier prevalence of 0.9% and a mortality rate of 0.9%.

**Meaning:**

In this study, most infections among health care workers occurred during the early stage of the COVID-19 outbreak and in low-contagion areas; routine screening may be helpful in identifying asymptomatic carriers.

## Teaser

This case series explores infection risk and clinical characteristics of health care workers with coronavirus disease (COVID-19) and discusses possible prevention measures.

## Introduction

The outbreak of coronavirus disease 2019 (COVID-19) first emerged in Wuhan, Hubei Province, China, in December 2019.^1,2,3,4^ Person-to-person transmission has been confirmed.^5,6,7^ On January 30, 2020, the World Health Organization declared a public health emergency of international concern.^8^ Although the government of Wuhan banned nonessential vehicles in the urban area, hospitals were still densely populated and became the most critical places to control the spread of COVID-19.

Unlike severe acute respiratory syndrome (SARS) or Middle East respiratory syndrome, COVID-19 was less virulent, with a lower mortality rate.^9,10,11^ Nevertheless, low virulence and longer incubation periods resulted in a significant number of asymptomatic carriers.^12^ These patients might not take adequate precautions and thus could become a source of transmission.^5^ Second-generation cases, spread by patients in the incubation period and asymptomatic carriers, have already been reported, and these cases appear to have included health care workers (HCWs).^13^ Asymptomatic transmission could further increase the risk of superspreading in hospitals.^14^ To date, research on transmission within hospitals has remained scarce.

Tongji Hospital is a large comprehensive tertiary hospital in Wuhan with more than 7000 beds and 9648 staff members. It was designated for the treatment of patients with COVID-19 during the epidemic outbreak. A total of 3110 of 9648 HCWs (32.2%) were transferred to fever clinics or wards to treat patients with the virus. By February 9, Tongji Hospital had received a total of 10 830 outpatients in fever clinics, with many later diagnosed with COVID-19. Frontline HCWs could be at high risk of infection because of close contact with these patients. Moreover, HCWs with infection could cause secondary transmission among patients, family members, and the community. Therefore, it is important to investigate the infection risk of HCWs and the clinical characteristics of affected cases.

Tongji Hospital took measures to protect HCWs with a risk-stratified strategy for prevention and control. Nevertheless, the rate of asymptomatic infection among HCWs and the scope of hospital environmental surface contamination were unknown. Identification of HCWs with infection and assessment of environmental surface contamination were crucial steps in tracking and halting the spread of SARS coronavirus 2 (SARS-CoV-2). This study aimed to evaluate the infection risk because of occupational exposure among HCWs and the clinical characteristics of affected HCWs. The effectiveness of possible prevention measures for first-line and non–first-line HCWs is also discussed.

## Methods

### Study Design and Participants

We retrospectively recruited 110 HCWs with COVID-19 at Tongji Hospital from January 1 to February 9, 2020. Exposure, epidemiologic, demographic information was retrospectively collected by a structured questionnaire, and the clinical, laboratory, and radiologic information was collected from electronic medical records. First-line HCWs were defined as those who worked in fever clinics or wards and provided direct care to patients with confirmed or suspected COVID-19. Non–first-line HCWs were defined as those who attended patients in general (ie, patients without COVID-19).

To detect the prevalence of subclinical infection of asymptomatic HCWs, we actively screened 335 HCWs at Tongji Hospital. The screened HCWs comprised 135 staff members (40.3%) from fever clinics or wards and 200 staff (59.7%) from other departments. Participants were randomly selected according to their staff identification numbers. The study was approved by the Tongji Hospital research ethics committee. Written informed consent was obtained from all participants. All collected data were securely stored in a database. This study followed the Strengthening the Reporting of Observational Studies in Epidemiology (STROBE) reporting guideline.

### Personal Protective Equipment and Environmental Cleansing Protocols

The personal protective equipment (PPE) used in low-contagion areas included surgical masks (equivalent to ASTM level 2), latex gloves and gowns (equivalent to AAMI level 2), and disposable round caps. The PPE used in the high-contagion areas included fit-tested particulate respirators (equivalent to an N95 mask), long-sleeved gowns (equivalent to AAMI level 4), goggles, disposable round caps, latex gloves, and shoe covers. The detailed environmental cleaning protocols were as follows: (1) a chlorine dioxide air disinfection machine was used 4 times a day for 2 hours at a time for air disinfection in wards with patients; (2) empty wards were irradiated with UV light once a day for 1 hour; (3) chlorine dioxide (500 mg/L) was sprayed with an ultra-low volume sprayer for air disinfection in public areas, with a dose of 20 to 30 mL/m^3^; and (4) surfaces of environmental objects were wrapped with chlorine-containing disinfection solution (1000 mg/L) twice a day. The environmental samples were collected after disinfection.

### Sampling Process

SARS-CoV-2 laboratory tests followed the World Health Organization recommendations.^15^ Nasopharyngeal swabs were obtained by nurses using a standard technique of wiping a flocculated swab across the posterior oropharynx from 1 tonsillar area to the other. Swabs were immediately placed in a transport medium, placed in a portable cooler, and delivered to our central laboratory.

Standard procedures^16^ were followed to collect environmental surface samples from fever clinics (n = 30), other departments (n = 30), and administration offices (n = 30). Sterile, cotton-tipped swabs prewetted with phosphate-buffer saline were drawn back and forth once across a 100-cm surface. Circular sweeps were used to swab objects with which individuals with infection would have come in contact, including diagnostic tables, door handles, bed bars, elevator buttons, computer keyboards and mouses, and registration machines, among others. After sampling, cotton rods were directly immersed in a phosphate-buffered saline solution and delivered to our central laboratory. Laboratory confirmation of COVID-19 was carried out by real-time reverse transcription–polymerase chain reaction using methods described previously.^17^

### Statistical Analysis

Continuous variables were described with medians and interquartile ranges (IQRs). Categorical variables were described as frequency and percentages. The Mann-Whitney U test, χ^2^ test, and Fisher exact test were used according to variable types as appropriate. The Poisson regression model was used to calculate the incident rate ratio (IRR) and 95% CIs for HCWs with COVID-19. A 2-sided *P* < .05 was considered statistically significant. All analyses were performed using SPSS statistical software version 20.0 (IBM Corp).

## Results

### Demographic Characteristics

By February 9, 2020, 2009 patients were diagnosed with COVID-19 in Tongji Hospital, 110 (5.5%) of whom were HCWs. Overall, 79 (71.8%) of those HCWs with infection were women, with a median (IQR) age of 36.5 (30.0-47.0) years. Most of this cohort were nurses (62 [56.4%]) and physicians (26 [23.6%]), and the remaining 22 (20.0%) were health care assistants. Of the 110 cases, 17 (15.5%) worked in fever clinics or wards, 73 (66.4%) worked in other clinical departments, and 20 (18.2%) worked in the hospital but did not interact with patients directly (Table 1). Poisson regression analysis showed that being younger than 45 years compared with older than 45 years, being a nurse compared with being a physician, and working in other clinical departments than fever clinics or wards were associated with increased risk of infection (<45 years: IRR, 1.9; 95% CI, 1.3-3.0; *P* = .002; nurses: IRR, 2.7; 95% CI, 1.7-4.3, *P* < .001; other clinical departments: IRR 3.1; 95% CI, 1.8-5.2, *P* < .001). The cumulative incidence of HCWs infected with COVID-19 in Tongji Hospital was 1.1% (110 of 9684). The infection rates were 0.5% among first-line HCWs (17 of 3110), 1.6% (73 of 4433) among HCWs in other clinical departments, and 1.0% (20 of 2012) among HCWs in departments with no contact with patients. The incidence of infection was significantly lower among first-line HCWs than that of the other HCW groups (0.5% [17 of 3110] vs 1.6% [93 of 6574]; *P* < .001).

**Table 1.** Basic Information for 9648 HCWs and 110 HCWs With Etiologically Confirmed COVID-19

Characteristic	No. (%)			IRR (95% CI)^a^	*P* value^b^	Estimated cumulative incidence (n = 9648), %	Proportion of all COVID-19 cases in hospital (n = 2009), %
	HCWs (N = 9648)	HCWs with COVID-19 (n = 110)	HCWs without COVID-19 (n = 9538)				
Age, y						1.1	5.5
≥45	1134 (11.8)	32 (29.1)	1102 (11.6)	1 [Reference]	NA	2.8	1.6
<45	8514 (88.2)	78 (70.9)	8436 (88.4)	1.9 (1.3-3.0)	.002	0.9	3.9
Sex							
Men	2550 (26.4)	31 (28.2)	2519 (26.4)	1 [Reference]	NA	1.2	1.5
Women	7098 (73.6)	79 (71.8)	7019 (73.6)	1.6 (1.01-2.4)	.04	1.1	3.9
Job category							
Physician	2151 (22.3)	26 (23.6)	2125 (22.2)	1 [Reference]	NA	1.2	1.3
Nurse	4417 (45.8)	62 (56.4)	4355 (45.7)	2.7 (1.7-4.3)	<.001	1.4	3.1
Health care assistant	3080 (31.9)	22 (20.0)	3058 (32.1)	0.8 (0.4-1.4)	.38	0.7	1.1
Department							
Fever clinic or ward	3110 (32.2)	17 (15.5)	3093 (32.4)	1 [Reference]	NA	0.5	0.8
Other clinical department^c^	4506 (46.7)	73 (66.4)	4433 (46.5)	3.1 (1.8-5.2)	<.001	1.6	3.6
Department with no patient contact^d^	2032 (21.1)	20 (18.2)	2012 (21.1)	1.2 (0.6-2.2)	.63	1.0	1.0

Abbreviations: COVID-19, coronavirus disease 2019; HCWs, health care workers; IRR, incident rate ratio; NA, not applicable.

^a^ Poisson regression was used to calculate IRRs and 95% CIs.

^b^ *P* values indicate differences between HCWs with COVID-19 and HCWs without COVID-19, from the result of a χ^2^ test.

^c^ These refer to clinical departments for patients presumed not to have COVID-19.

^d^ These refer to the logistics and administrative departments in the hospital, which usually have no direct contact with patients.

### Development of the Epidemic in HCWs

The Figure shows the dynamic change of COVID-19 cases in Wuhan and among HCWs in Tongji Hospital by date of illness onset. Compared with the infection rate of 0.18% (16 903 of 9 083 500) in Wuhan (estimated on February 9, 2020), the infection rate among medical staff in Tongji Hospital was higher (1.1% vs 0.18%; *P* < .001). A risk-stratified strategy for prevention and control was strictly performed in Tongji Hospital to curb transmission, such as immediate isolation of HCWs with clinically confirmed and suspected cases of COVID-19, release of infection prevention and control guidelines, and training in infection control measures, including both face-to-face training and mobile messages. Furthermore, 2 rounds of surveillance per day were established to monitor HCWs’ compliance with standard precautions. The curve of HCWs with new infections per day reached its peak on January 20, 2020. It decreased dramatically on January 28, 2020, and remained low (≤3 per day) after February 1, 2020. However, the number of newly confirmed cases of COVID-19 in Wuhan continued to increase after February 1 (Figure).

**Figure.**
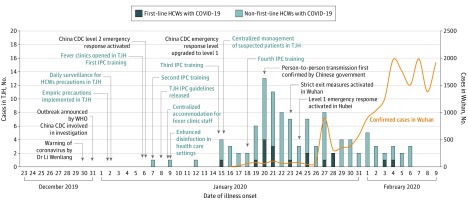
Change of Daily Infected Numbers of Health Care Workers (HCWs) With Coronavirus Disease 2019 (COVID-19) in Tongji Hospital (TJH) Daily numbers of HCWs who were infected with severe acute respiratory syndrome coronavirus 2 (SARS-CoV-2) in TJH by date of illness onset. The decline in incidence after January 20 is likely because of a risk-stratified strategy of prevention and control. China CDC indicates Chinese Center for Disease Control and Prevention; and IPC, infection and protection control.

### Exposure Information

According to reports by 110 HCWs with COVID-19, 70 (63.6%) were presumably infected in general clinics or wards, 7 (6.4%) in fever clinics or wards, and 14 (12.7%) through community-acquired infection. A total of 66 (60.0%) reported disease onset on or before January 20 and 25 (22.7%) after January 20; 65 (59.1%) attributed infection to contact with patients who were later diagnosed with COVID-19, 12 (10.9%) to contact with colleagues, and 14 (12.7%) to contact with family or friends. The other 19 HCWs (17.3%) could not recall their exposure history. None had been to Huanan seafood market, but 13 (11.8%) had been to other wet markets. Regarding the type of onset, 31 (28.2%) were clustered, and 79 (71.8%) were independent cases. Overall, 40 HCWs (36.4%) reported that they had transmitted the virus to their family or friends.

The exposure location of first-line HCWs was different from that of non–first-line HCWs. More first-line HCWs than non–first-line HCWs got infected in fever clinics or wards (7 [41.2%] vs 0) but fewer in general wards or clinics (6 [35.3%] vs 64 [68.8%]) (*P* < .001). Non–first-line nurses younger than 45 years were more likely to be infected compared with first-line physicians aged 45 years or older (IRR, 16.1; 95% CI, 7.1-36.3; *P* < .001). There was no difference between first-line and non–first-line HCWs on other survey items associated with exposure (Table 2).

**Table 2.** Exposure Information of 110 First-Line and Non–First-Line HCWs With Coronavirus Disease 2019

Characteristic	HCWs, No. (%)			*P* value^a^
	All (N = 110)	First-line		
		Yes (n = 17)	No (n = 93)	
Age, median (IQR), y	36.5 (30.0-47.0)	36.0 (32.0-41.0)	37.0 (30.0-47.0)	.88
Sex				
Men	31 (28.2)	8 (47.1)	23 (24.7)	.08
Women	79 (71.8)	9 (52.9)	70 (75.3)	
Job category				
Physician	26 (23.6)	6 (35.3)	20 (21.5)	.35
Nurse	62 (56.4)	7 (41.2)	55 (59.1)	
Health care assistant	22 (20.0)	4 (23.5)	18 (19.4)	
Presumed exposure location				
General wards or clinics	70 (63.6)	6 (35.3)	64 (68.8)	<.001
Fever wards or clinics	7 (6.4)	7 (41.2)	0	
Not in hospital	14 (12.7)	1 (5.9)	13 (14.0)	
Unknown	19 (17.3)	3 (17.6)	16 (17.2)	
General wards or clinics vs fever wards or clinics	NA	NA	NA	<.001
Presumed exposure time				
January 20 and before	66 (60.0)	13 (76.5)	53 (57.0)	.18
After January 20	25 (22.7)	1 (5.9)	24 (25.8)	
Unknown	19 (17.3)	3 (17.6)	16 (17.2)	
Identified exposure to confirmed cases				
Patients	65 (59.1)	12 (70.6)	53 (57.0)	.65
Colleagues	12 (10.9)	1 (5.9)	11 (11.8)	
Family or friends	14 (12.7)	1 (5.9)	13 (14.0)	
Unknown	19 (17.3)	3 (17.6)	16 (17.2)	
Other exposure				
Exposure to Huanan seafood market	0	0	0	.25
Exposure to other wet market	13 (11.8)	0	13 (14.0)	
No exposure to wet market	78 (70.9)	14 (82.4)	64 (68.8)	
Unknown	19 (17.3)	3 (17.6)	16 (17.2)	
Type of onset				
Clustered	31 (28.2)	4 (23.5)	27 (29.0)	.78
Diffused	79 (71.8)	13 (76.5)	66 (71.0)	
Transmitted to family or friends	40 (36.4)	7 (41.2)	33 (35.5)	.79
Prevalence of asymptomatic infection, No./total No. (%)^b^	3/335 (0.9)	1/135 (0.7)	2/200 (1.0)	.81

Abbreviations: HCWs, health care workers; IQR, interquartile range; NA, not applicable.

^a^ *P* values indicate differences between first-line HCWs and non–first-line HCWs from χ^2^ test (for rate) and U test (for age).

^b^ Data are No./total No. (%) for, where total No. represents the patients and samples with available data.

To explore the prevalence of possible asymptomatic infection in HCWs, we selected 335 staff members randomly according to their employee identification numbers for SARS-CoV-2 reverse transcription–polymerase chain reaction tests. The screened HCWs were composed of 135 (40.3%) first-line staff and 200 (59.7%) non–first-line staff. Among the 135 staff in fever clinics or wards, 1 physician (0.79%) had positive test results (Table 2). This physician was assigned to work in fever clinics once with appropriate PPE. They were asymptomatic, and no infective lesions were found by radiologic investigation.

Of the 200 screened staff in other clinical departments, 1 oncologist and 1 member of the security staff (1.0%) had positive test results for COVID-19 (Table 2). The oncologist claimed no symptoms, and ground-glass opacities were later identified in the lower lobe of their right lung by computed tomography (CT) scan. The other case was a security captain of the outpatient department who had no direct interaction with patients but was infected by a friend later confirmed to have COVID-19. This patient’s CT scan showed no sign of infection.

Potential virus contamination and airborne dispersal in the health care settings in Tongji Hospital were tested. A total of 90 samples were collected from environmental surfaces, such as diagnostic tables, door handles, bed bars, elevator buttons, computer keyboards and mouses, and registration machines in fever clinics and wards (n = 30), other clinical departments (n = 30), and the administration office rooms (n = 30). No surface specimen tested positive (Table 2).

### Clinical Characteristics of HCWs With COVID-19

Clinical characteristics of 110 confirmed HCWs with COVID-19 are shown in Table 3. The rate of comorbidities in affected HCWs was 12.7% (14 of 110). The 4 most common comorbidities among all HCWs with infection were hypertension (12 [10.9%]), cardiovascular disease (3 [2.7%]), chronic obstructive pulmonary disease (2 [1.8%]), and chronic liver disease (2 [1.8%]). The 5 most common symptoms were fever (67 [60.9%]), myalgia or fatigue (66 [60.0%]), cough (62 [56.4%]), sore throat (55 [50.0%]), and muscle ache (50 [45.5%]). Regarding disease severity, most HCWs had nonsevere disease (93 [84.5%]; 31 [28.2%] with mild disease and 62 [56.2%] with moderate disease), while 12 (10.9%) had severe disease, 4 (3.6%) had critical disease, and 1 (0.9%) died. The median (IQR) incubation period was 5 (3-8) days, and the median (IQR) time from onset of symptom to treatment was 1 (0-2) days. There was no difference between first-line and non–first-line HCWs in the subgroups of comorbidity and common symptoms.

**Table 3.** Clinical Characteristics of 110 HCWs With Etiologically Confirmed Coronavirus Disease 2019

Characteristic	HCWs, No. (%)			*P* value^a^
	All (N = 110)	First-line		
		Yes (n = 17)	No (n = 93)	
Any comorbidity	14 (12.7)	1 (5.9)	13 (14.0)	.69
Hypertension	12 (10.9)	1 (5.9)	11 (11.8)	.69
Cardiovascular disease	3 (2.7)	0	3 (3.2)	>.99
COPD	2 (1.8)	0	2 (2.2)	>.99
Chronic liver disease	2 (1.8)	0	2 (2.2)	>.99
Chronic kidney disease	1 (0.9)	0	1 (1.1)	>.99
Diabetes	1 (0.9)	0	1 (1.1)	>.99
Tuberculosis	1 (0.9)	0	1 (1.1)	>.99
Cerebrovascular disease	0	0	0	NA
Malignant neoplasm	0	0	0	NA
Signs and symptoms				
Fever	67 (60.9)	7 (41.2)	60 (64.5)	.10
Myalgia or fatigue	66 (60.0)	12 (70.6)	54 (58.1)	.42
Cough	62 (56.4)	9 (52.9)	53 (57.0)	.80
Sore throat	55 (50.0)	9 (52.9)	46 (49.5)	>.99
Muscle ache	50 (45.5)	7 (41.2)	43 (46.2)	.79
Diarrhea	39 (35.5)	4 (23.5)	35 (37.6)	.41
Headache	33 (30.0)	5 (29.4)	28 (30.1)	>.99
Dyspnea	26 (23.6)	4 (23.5)	22 (23.7)	>.99
Dizziness	24 (21.8)	5 (29.4)	19 (20.4)	.52
Sputum production	16 (14.5)	3 (17.6)	13 (14.0)	.71
Nausea and vomiting	15 (13.6)	2 (11.8)	13 (14.0)	>.99
>1 sign or symptom	15 (13.6)	2 (11.8)	13 (14.0)	>.99
Fever, cough, and dyspnea	13 (11.8)	1 (5.9)	12 (12.9)	.69
Hemoptysis	1 (0.9)	0	1 (1.1)	>.99
Disease severity				
Mild	31 (28.2)	7 (41.2)	24 (25.8)	.66
Moderate	62 (56.2)	8 (47.1)	54 (58.1)	
Severe	12 (10.9)	1 (5.9)	11 (11.8)	
Critical	4 (3.6)	1 (5.9)	3 (3.2)	
Fatal	1 (0.9)	0	1 (1.1)	
Mechanical ventilation required	4 (3.6)	1 (5.9)	4 (4.3)	.58
Incubation period, median (IQR), d	5.0 (3.0-8.0)	7.0 (2.3-9.5)	5.0 (3.0-7.0)	.50
Onset of symptom to treatment, median (IQR), d	1.0 (0-2.0)	1.0 (0-3.0)	1.0 (0-2.0)	.59

Abbreviations: COPD, chronic obstructive pulmonary disease; HCWs, health care workers; IQR, interquartile range; NA, not available.

^a^ *P* values indicate differences between first-line HWS and non–first-line HWS, from the result of χ^2^ test and *t* test.

Table 4 shows the laboratory findings for HCWs with COVID-19 on admission to hospital. The routine blood examination indicated increased erythrocyte sedimentation (median [IQR], 19 [9-39] mm/h), but blood counts and other indicators were within reference range. Erythrocyte sedimentation in non–first-line HCWs was higher than first-line HCWs (19.0 [9.0-40.5] mm/h vs 10.0 [4.5-24.5] mm/h; *P* = .04). Other biochemical examinations showed higher than reference levels of interleukin-2 receptor (reference range: 0.1-4.1 pg/mL; all HCWs with COVID-19: median [IQR], 463.5 [368.8-660.3] pg/mL), interleukin-6 (reference range: 0.1-2.9 pg/mL; all HCWs with COVID-19: median [IQR], 12.7 [4.0-24.6] pg/mL), C-reactive protein level (reference range, <0.1 mg/L; all HCWs with COVID-19: median [IQR], 0.94 [0.30-2.93] mg/dL [to convert to milligrams per liter, multiply by 10.0]), and fibrin (reference range: 2.00-4.00 g/L; all HCWs with COVID-19: median [IQR], 4.12 [3.59-4.95] g/L). Interleukin-6 levels were significantly higher in non–first-line HCWs than first-line HCWs (14.9 [5.3-26.5] pg/mL vs 3.8 [1.5-12.1] pg/mL; *P* = .04).

**Table 4.** Laboratory and CT Indicators for HCWs With Coronavirus Disease 2019 on Admission to Hospital

Indicator	Reference range	HCWs, Median (IQR)			*P* value^a^
		All	First-line		
			Yes	No	
**Laboratory test**					
White blood cell count, /μL	3500-9500	4850 (3870-6170)	4730 (3780-5970)	4.85 (3.87-6.28)	.84
Neutrophil count, /μL	1800-6300	2390 (3000-3930)	3090 (2070-4180)	2970 (2400-3930)	.96
Lymphocyte count, /μL	1100-3200	1140 (860-1610)	1230 (810-1600)	1130 (860-1620)	.78
Monocyte count, ×10^9^/L	100-600	410 (300-530)	360 (310-560)	410 (300-540)	.88
Red blood cell count, ×10^6^/μL	4.30-5.80	4.39 (4.04-4.70)	4.50 (4.31-4.72)	4.34 (4.00-4.70)	.14
Platelet count, ×10^3^/μL	125.0-350.0	188.0 (154.5-238.8)	186.0 (157.5-236.5)	188.0 (154.0-239.0)	.92
Hemoglobin, g/dL	11.5-15.0	12.7 (11.5-14.1)	12.8 (6.2-14.)	12.7 (11.6-13.8)	.70
Erythrocyte sedimentation, mm/h	0-15	19 (9-39)	10.0 (4.5-24.5)	19.0 (9.0-40.5)	.04
Ferritin, ng/mL	30-400	293.7 (118.3-622.8)	261.0 (56.4-793.5)	330.4 (118.3-622.8)	.68
Interleukin-1α, pg/mL	<5	5 (5-5)	5 (5-5)	5 (5-5)	.28
Interleukin-2 receptor, pg/mL	0.1-4.1	463.5 (368.8-660.3)	447.0 (339.0-1028.0)	480.0 (371.5-654.5)	.79
Interleukin-6, pg/mL	0.1-2.9	12.7 (4.0-24.6)	14.9 (5.3-26.5)	3.8 (1.5-12.1)	.04
Interleukin-8, pg/mL	<62	13.7 (8.8-23.4)	7.0 (6.6-20.0)	14.4 (9.7-24.1)	.10
Interleukin-10, pg/mL	<9.1	5.0 (5.0-6.4)	5.0 (5.0-5.3)	5.0 (5.0-7.3)	.89
Tumor necrosis factor, pg/mL	<8.1	7.4 (6.1-9.2)	6.7 (5.3-7.1)	7.7 (6.3-9.5)	.08
Procalcitonin, ng/mL	0.02-0.05	0.04 (0.02-0.06)	0.05 (0.03-0.08)	0.04 (0.02-0.06)	.40
C-reactive protein level, mg/dL	<0.1	0.94 (0.30-2.93)	0.80 (0.04-2.41)	0.97 (0.32-3.14)	.45
Aminotransferase, U/L					
Aspartate	≤40	16 (11-23)	21.0 (18.0-32.5)	23.0 (17.0-33.0)	.55
Alanine	≤41	22 (18-32)	18.0 (11.5-27.0)	16.0 (11.0-22.5)	.94
Lactate dehydrogenase, U/L	135-225	215.5 (180.75-279.25)	199.0 (174.0-271.8)	218.0 (185.5-279.8)	.50
Total bilirubin, mg/dL	≤1.52	0.39 (0.29-0.59)	0.36 (0.27-0.58)	0.40 (0.29-0.60)	.65
Glucose, mg/dL	74.05-109.00	103.24 (98.20-119.82)	112.25 (98.74-137.12)	103.24 (97.84-118.74)	.42
Albumin, g/dL	3.50-5.20	4.13 (3.84-4.40)	4.18 (4.02-4.31)	4.03 (3.83-4.40)	.53
Creatinine, mg/dL	0.67-1.18	0.67 (0.61-0.88)	0.79 (0.62-0.95)	0.67 (0.61-0.87)	.42
Glomerular filtration rate, mL/min/1.73m^2^	>90	106.4 (88.4-118.6)	104.9 (78.1-117.3)	1.6.7 (89.4-118.8)	.93
BUN, mmol/L	2.6-7.5	3.8 (3.1-4.8)	4.2 (3.3-5.0)	3.6 (3.0-4.8)	.47
Prothrombin time, s	11.5-14.5	13.6 (12.9-14.3)	13.0 (12.8-13.4)	13.7 (12.9-14.4)	.18
International normalized ratio	0.80-1.20	1.04 (0.97-1.09)	0.97 (0.96-1.02)	1.05 (0.97-1.10)	.16
Fibrin degradation product, μg/mL	<5.0	4.0 (4.0-4.0)	4.0 (4.0-4.0)	4.0 (4.0-4.0)	.86
Fibrin, g/L	2.00-4.00	4.12 (3.59-4.95)	3.95 (2.91-4.84)	4.12 (3.64-4.95)	.41
D-dimer, μg/mL	<0.5	0.45 (0.26-0.66)	0.50 (0.22-0.63)	0.45 (0.27-0.66)	.81
Cardiac troponin I, ng/mL	≤0.034	0.028 (0.019-0.047)	0.019 (0.019-0.037)	0.028 (0.019-0.063)	.17
Brain-type natriuretic peptide, pg/mL	<486	26.0 (11.5-62.0)	16.5 (8.0-31.3)	28.0 (13.0-77.0)	.17
Total amylase, U/L	28-100	54.5 (39.0-81.5)	70.0 (42.0-83.0)	54.0 (36.0-81.0)	.55
Uric acid, mg/dL	2400.0-5700.8	3991.6 (3467.2-5401.2)	4423.5 (3806.7-5847.1)	3983.2 (3458.8-4890.8)	.24
**CT scan, No. (%)**					
Area affected					
Unilateral involved	NA	29 (26.4)	3 (17.6)	26 (27.9)	.57
Bilateral involved	NA	49 (44.5)	4 (23.5)	45 (48.4)	.73
Lung lobes involved					
Single	NA	26 (23.6)	3 (17.6)	23 (24.7)	.37
Multiple	NA	52 (47.3)	4 (23.5)	48 (51.6)	.50
Ground-glass opacity	NA	45 (40.9)	4 (23.5)	41 (44.1)	.21
Consolidation	NA	26 (23.6)	2 (11.8)	24 (25.8)	.70
White lung	NA	2 (1.8)	0	2 (2.2)	.51

Abbreviations: BUN, blood urea nitrogen; CT, computed tomography; D-dimer, dimerized plasmin fragment D; HCWs, health care workers; IQR, interquartile range.

SI conversion factors: To convert alanine aminotransferase to microkatals per liter, multiply by 0.0167; amylase to microkatals per liter, muliply by 0.0167; aspartate aminotransferase to microkatals per liter, multiply by 0.0167; brain-type natriuretic peptide to nanograms per liter, multiply by 1.0; cardiac troponin I to micrograms per liter, multiply by 1.0; C-reactive protein to milligrams per liter, multiply by 10.0; creatine kinase to microkatals per liter, multiply by 0.0167; creatinine to micromoles per liter, multiply by 88.4; D-dimer to nanomoles per liter, multiply by 5.476; ferritin to micrograms per liter, multiply by 1.0; fibrin degradation product to milligrams per liter, multiply by 1.0; glucose to micromoles per liter, multiply by 0.0555; hemoglobin to grams per liter, multiply by 10.0; lactate dehydrogenase to microkatals per liter, multiply by 0.0167; neutrophil count, lymphocyte count, monocyte count, and white blood cell count to ×10^9^ per liter, multiply by 0.001; platelet count to ×10^9^ per liter, multiply by 1.0; red blood cell count to ×10^12 ^per liter, multiply by 1.0; total albumin to grams per liter, multiply by 10.0; total bilirubin to micromoles per liter, multiply by 17.104; and uric acid to micromoles per liter, multiply by 0.0595.

^a^ *P* values indicate differences between first-line HWS and non–first-line HWS, from the result of *t* test.

Chest CT findings are shown in Table 4. Of 110 HCWs with COVID-19, 78 (70.9%) showed lesions on chest CT, with 29 (26.4%) unilateral involvement and 49 (44.5%) bilateral involvement. A total of 26 (23.6%) showed infection on single lung lobes, and 52 (47.3%) had multiple lung lobes affected. The other 31 HCWs (29.1%) had no obvious lesions on CT. The numbers of cases with features of ground-glass opacity, consolidation, and white lung were 46 (41.8%), 26 (23.6%), and 2 (1.8%), respectively.

## Discussion

Our study was based in a large comprehensive tertiary hospital in Wuhan, China. As a large number of outpatients were treated in fever clinics every day, HCWs were at high infection risk. Superspreading events had been reported in other hospitals.^14^ Compared with the infection rate of 0.18% in Wuhan (estimated February 9, 2020), the infection rate of 1.1% among medical staff in Tongji Hospital was much higher. Moreover, our results showed that non–first-line HCWs had a significantly higher infection rate compared with first-line HCWs. This can be explained by several reasons. First, unlike SARS and Middle East respiratory syndrome, the incubation time of COVID-19 is much longer.^18^ This made it hard to recognize patients with the disease at an early stage. Insufficient protective measures were available in clinical departments other than fever clinics and wards, which could have put non–first-line HCWs at a higher risk. Our results indicated that most HCWs (60.0%) were infected during the early stage of the COVID-19 outbreak. Second, the virulence of SARS-CoV-2 may not be as severe as SARS.^2^ Many patients who were infected had no or very subtle symptoms, and some exhibited atypical symptoms.^19^ The existence of such patients could greatly endanger the health of staff even though clinical areas caring for patients with and without COVID-19 were separated from each other. Third, because of lack of disease knowledge, it was difficult to identify patients with COVID-19 at the beginning of this epidemic.

A total of 0.9% of HCWs with COVID-19 were asymptomatic. Until now, little was known about the risk of transmission from asymptomatic carriers. One reason for the rapid spread worldwide could be asymptomatic patients in the early stage.^5^ The viral load detected in asymptomatic patients was similar to that detected in symptomatic patients, indicating the transmission potential of asymptomatic carriers of SARS-CoV-2.^12^ These asymptomatic HCWs might become a risk factor for patients, colleagues, and the community. Therefore, identification of asymptomatic carriers among HCWs would be important, and asymptomatic carriers should be isolated from family and colleagues to avoid cross-infection. Control of transmission in HCWs could depend on maintaining a low threshold for suspicion of infection. Certain signs may indicate that staff should be tested, including having worked at or attended a health care facility in the past 14 days where more than 2 patients with hospital-associated COVID-19 infections have been reported, symptoms of SARS (ie, fever, cough, or requiring admission to hospital), or close contact with a confirmed or suspected case of COVID-19 in the past 14 days. Staff should be tested twice, when they transfer from the fever clinic and 14 days after. Staff with positive results should be treated immediately.

In our study, most infections in HCWs occurred at the early stage of the epidemic, before protective measures were taken. This may imply that the rigorous prevention measures taken in Tongji Hospital were effective to prevent or limit transmission in a health care setting. We stressed the importance of appropriate use and especially disposal of PPE for HCWs later in the outbreak to ensure that PPE was effective and to avoid any increase in transmission. Moreover, we strictly divided the highly contagious areas (ie, fever clinics and wards) in 3 parts, as clean areas, potentially contaminated areas, and contaminated areas. Furthermore, we separated medical staff and patient access to avoid possible cross-infection. No surface specimen tested positive. A possible explanation is that virus contamination in the health care setting might not be a source of transmission to patients or of nosocomial outbreaks for COVID-19. Moreover, cleaning and disinfection procedures, which were ensured consistently and correctly in the hospital, could largely reduce the spread of many pathogens in the health care setting.^20,21^ Cleaning surfaces with water and detergent and applying additional hospital disinfectants (such as chlorine dioxide disinfectant) could be an effective and sufficient procedure to prevent transmission of COVID-19 through environmental contamination.

In this study, 84.5% of affected HCWs had mild or moderate disease. There are several possible reasons for this. First, most affected HCWs in our study were young adults. Patients with severe and critical COVID-19 are usually older. Second, early symptoms were more easily noticed by HCWs, which could also explain the lower frequency of fever reported in our study. Fever was much more common in other studies, including among patients with severe COVID-19.^13^ Third, the time between symptom onset and treatment was 1 day, which was much shorter than in other studies.^19,22^ All these may indicate that early diagnosis and treatment favored a better outcome for patients with COVID-19. Half the HCWs with infection were treated outside the hospital. This might indicate that treating mild patients outside a hospital setting with appropriate guidance from qualified medical professionals could be a feasible method when hospital capacity is limited.

### Limitations

This study has limitations. The recall bias of this survey could be a concern, but information collection took place recently, and the possibility of recall bias was small. Furthermore, the information collected was about concrete behaviors, especially when the surveyed subjects were HCWs.

## Conclusions

In this study, non–first-line HCWs were at a high risk of infection during the early stage of the COVID-19 outbreak, and interventions targeting this group should be evaluated. Most HCWs with infection had mild symptoms; however, special attention needs to be paid to protect HCWs from cross-infection from other HCWs.
